# Chitosan processing waste nutrients compounds as a potential natural poultry premix

**DOI:** 10.5455/javar.2025.l878

**Published:** 2025-03-24

**Authors:** Rosa Tri Hertamawati, Shokhirul Imam, Reikha Rahmasari, Ujang Suryadi

**Affiliations:** Department of Animal Science, Politeknik Negeri Jember, Jawa Timur, Indonesia

**Keywords:** Amino acid, mineral, nutrient, premix, supplement

## Abstract

**Objective:**

This study aimed to use the potential chitosan processing waste from shrimp heads as a premix to improve the production performance of laying chickens.

**Materials and Methods:**

This research uses shrimp head waste, NaOH, hydrochloric acid, Na_2_SeO_3,_ and NaCl solutions. Processing shrimp head waste into chitosan is done in three ways, namely demineralization, deproteination, and deacetylation. Each of the resulting liquid wastes is then mixed until a neutral mixture is obtained. The data observed were the nutrient and amino acid content of liquid waste from the deproteinization, demineralization, and deacetylation processes, as well as the neutral mixture.

**Results:**

The results showed liquid waste from the process of making chitosan from shrimp head waste using deproteination, demineralization, and deacetylation methods, as well as a neutral mixture containing little energy, protein, and fat but is rich in minerals. The most abundant and complete amino acid content is found in deproteination process wastewater and neutral mixtures.

**Conclusion:**

In conclusion, the waste from making chitosan from shrimp head waste through deproteinization, demineralization, and deacetylation, and the neutral mixture contains enough minerals and amino acids needed by livestock, so it has the potential to be used as a premix.

## Introduction

The quality and quantity of feed for laying hens during the production period is critical because it will affect livestock productivity, including the number and quality of eggs produced, and then influence the success of the business. Eggs have many important functions, including foaming, gel formation, and emulsification [1]. Based on their value and functional properties, eggs as a whole are the main ingredient in many food products.

The physical and chemical quality of eggs is related to the nutritional quality of the feed. Nutrient needs such as protein, energy, fat, crude fiber, minerals, and vitamins are basic needs that are sometimes not met in rations, especially in self-mixing rations. Therefore, the addition of a vitamin-mineral premix and an amino acid premix is very necessary. Premix has long been used as an important feed supplement for laying hens because it plays an important role in performance, health, production, and business success. Supplementation with a mineral and vitamin premix up to 0.45% on the laying hen diet during the first laying phase can increase the performance and decrease feed cost production [2]. Premixes circulating on the market have been prepared according to livestock needs, type of livestock, and production objectives. The addition of premix in the feed mixture can be given as much as 2%–10% [3].

Premixes produced commercially and circulating on the market are costly because they are made with commercial ingredients; however, to reduce manufacturing costs, premixes can be made using waste from the chitosan manufacturing process. Chitosan is a waste processing product from the shrimp processing industry that is exported in the form of frozen shrimp. The production of shrimp commodities captured by fisheries in Indonesia reached 206,772 tons (2020) and 247,506 tons (2021); for East Java, in 2020, it was 5,633 tons, and in 2021, it reached 14,439 tons, according to data from BPS -Statistics Indonesia. Shrimp caught in Indonesia with good quality are usually processed into frozen food (frozen shrimp) for export. Shrimp waste produced from frozen shrimp processing is estimated to reach 35%–70% of the weight of whole shrimp. The parts included in shrimp waste are heads, skin, and shrimp that are not suitable for consumption. The portion of whole shrimp that becomes waste from frozen shrimp processing, the head and tail, reaches 45%–60% of the total weight of shrimp [4]. The waste used in the chitosan industry is discarded heads, tails, and shells, and all this time the shrimp waste has only been used by the community around the shrimp processing factory as a mixture for making shrimp paste, shrimp crackers, or shrimp paste or can also be used as a mixture for animal feed. The process of making chitosan also produces liquid waste. If the liquid waste is disposed of without processing, it will hurt the environment. Environmental pollution caused by liquid waste from making chitosan includes physical and chemical changes in water, resulting in an impact on microfauna and microflora, high-concentration residues of nitrogen, phosphorus, organic carbon, suspended solids, and oxygen. Industrial wastewater containing various chemical compounds can harm the river systems [5,6]. Most of the chitosan processing liquid waste is used to make low-value products such as fertilizer and animal feed [7]. Based on that information, this study aimed to explore the potential of chitosan processing waste from shrimp heads as a premix to improve the production performance of laying chickens.

## Material and Methods

### Materials

The material used for the procedures was a total of 10 kg of shrimp head waste, which was obtained from the Muncar–Banyuwangi and Puger–Jember frozen shrimp processing industry.

### Procedures

The shrimp waste was obtained from the Puger and Muncar frozen shrimp processing industry. The shrimp head waste is then washed cleaned and dried. Next, the clean shrimp head waste is crushed by grinding. The first process in making chitosan is demineralization. The sample was weighed at 66.5 gm and then added with 1,000 ml of 1 M hydrochloric acid (HCl). The mixture was then heated at 60°C for 1 h and continued to stir. After that, the mixture is then filtered. The solid part (residue) is rinsed with distilled water until the pH is neutral. The demineralized solid is then dried in an oven at a temperature of 60°C–70°C for 1 h. The second stage is deproteination. The shrimp head waste solids were weighed at 21.1 gm and then added with 211 ml of 3.5% NaOH. The mixture was then heated at 70°C for 1 h while continuing to stir. The mixture is then filtered, and the deacetylation process continues.

### Nutritional evaluation analysis

Proximate analysis of deproteinized, demineralized, and deacetylated shrimp head waste filtrate, as well as a neutral mixture including water, ash, crude protein, crude fat, and carbohydrate content. Water content is measured based on SNI 01-2891-1992, point 5.1 (SIG 2023). Ash content was measured using a method based on SNI 01-2891-1992, point 6.1 (SIG 2023). Ash content was calculated by burning the samples in a furnace at a temperature of 550°C. Crude protein content analysis was carried out based on 18-8-31/MU/SMM-SIG (Titrimetry) (SIG 2023). Crude fat content was measured using a calculation method based on 18-8-5/MU/SMM-SIG point 3.3.3 (Weibull) (SIG 2023). Mineral content was measured at PT. Saraswanti Indo Genetech using the inductively coupled plasma–optical emission spectrometry (ICP-OES) method based on the 18-13-1/MU/SMM-SIG (ICP OES) method (SIG 2023). The minerals analyzed are calcium, potassium, and phosphorus. The amino acid content was analyzed at PT. Saraswanti Indo Genetech uses the ultra performance liquid chromatography (UPLC) method based on the 18-5-17/MU/SMM-SIG (UPLC-PDA) method.

### Statistical analysis

Data on nutrient content, minerals, and amino acid composition of waste from the process of making chitosan from shrimp heads were analyzed using the descriptive method to describe the existing bioactive components.

## Results and Discussion

### The proximate result

Nutrient content (crude protein, crude fat, ash, and carbohydrates) and energy composition of the time processing of shrimp waste at each step were presented in Table 1. The highest crude protein, ash, and carbohydrate content are in deproteination process waste, namely 1.86%, 4.69%, and 2.02%. Deproteination is the process of removing protein from shrimp head waste using NaOH [8]. The high protein content in liquid waste from the deproteination process is thought to be due to the dissolution of protein from shrimp head waste by NaOH. The deproteination stage in chitin isolation will cause the protein from shrimp head waste to dissolve in alkaline so that proteins that are covalently bound to the chitin functional groups will be separated [8]. NaOH added in the deproteination stage will release Na⁺ ions, which will bind to the protein and form sodium proteinate [9]. The highest protein content was also obtained in the deproteinization process of shrimp waste by fermentation by *Bacillus licheniformis* with an incubation time of 2 days (CP 47.19%) [10]. The protein content of each waste from the chitosan manufacturing process still has a protein content that has the potential to be used as a feed supplement and can be seen from the amino acid content.

**Table 1.** Results of proximate analysis and mineral content.

No	Parameters	Unit	Neutral	Distillation	Demineralization	Deproteination
1	Total energy	kcal/100 gm	85.6	80.4	81.8	155.2
2	Energy from fat	kcal/100 gm	0	0	0	0
3	Ash	%	3.49	4.38	3.04	4.695
4	Moisture	%	94.37	93.61	94.915	91.425
5	Carbohydrate	%	0.395	1.205	1.83	2.025
6	Ether extract	%	<0.02	<0.02	<0.02	<0.02
7	Crude protein	%	1.745	0.805	0.215	1.855
8	Potassium	mg/100 gm	51.015	5.095	3.02	70.79
9	Phosphorus	mg/kg	484.285	41.885	1,478.565	172.885
10	Calcium	mg/100 gm	235.375	4.935	801.095	56.36

The ash content indicates the presence of inorganic compounds and is related to the mineral content in the waste solution from each process of making chitosan and the neutral mixture. The ash content of shrimp head waste is approximately 24.42%–33.46% [11,12]. From each demineralization, deproteinization, deacetylation, and neutral mixture process waste, the ash content is 3.04%, 4.69%, 4.38%, and 3.49%. These results indicate that the three stages of the chitosan manufacturing process can dissolve the ash in shrimp head waste. The ash content of chitin is influenced by the HCl concentration and heating temperature in the demineralization process. The higher the HCl concentration and temperature given, the lower the ash content in chitin (yield). The greater the protein and ash lost from chitin, the purer the chitin produced [13].

The highest to lowest carbohydrate content of the chitosan manufacturing process waste is deproteination, demineralization, distillation/deacetylation, and neutral mixture. The respective carbohydrate content is 2.02%, 1.83%, 1.20%, and 0.39%. During the chitosan manufacturing process, particularly in the deproteinization and demineralization steps. The carbohydrate content may not be entirely removed from the shrimp waste depending on the types of carbohydrates and crustacean shells. The fat content of the four solutions showed no different results; it was less than 0.02%. The low-fat content of the four solutions may be due to the low-fat content of shrimp shells. The fat content in shrimp shells is around 2.47% [14]. Several factors can influence the fat content in shrimp heads and shells. The fat composition from aquatic organisms is dependent on feed, size, age, and environmental conditions [15]. The reduction in protein and fat levels in the process of making chitosan is due to the use of hydrochloric acid and sodium hydroxide which are strong acids and strong bases that can dissolve fat and protein [15].

### Mineral results

Mineral data from the analysis of waste from the chitosan manufacturing process is shown in Table 1. Mineral content is usually associated with the ash content of a material because minerals are a fraction of inorganic compounds. The types of minerals measured are potassium, phosphorus, and calcium minerals. The analysis results show that the highest potassium mineral content is in the deproteination process waste (70.79 mg/100 gm). The highest phosphorus and calcium mineral content is in the demineralization process waste, 1,478.56 mg/kg and 801.09 mg/100 gm.

Demineralization is the process of removing minerals from shrimp head waste using an acid solution such as HCl, HNO_₃_, CH_3_COOH, HCOOH, and H_2_SO_4_, minerals that may be found are calcium, phosphorus, and magnesium [16,17]. The high calcium mineral content in demineralized waste is because calcium carbonate (CaCO_3_), which is the main mineral in shrimp head waste, is dissolved in the HCl acid solution. Calcium compounds in demineralization react to produce calcium chloride, which is soluble in water, CO_2_ gas, and HCl solution. The amount of minerals that dissolve in the demineralization process is influenced by several factors, including the concentration of the solvent and the length of the soaking process. CaCO_3_ levels in ebi shells decreased as the HCl concentration increased.

Calcium and phosphorus are essential minerals in poultry and livestock diet formulations [18]. Both minerals are correlated with bone and eggshell health. Calcium has an important role in poultry diets, as 99% of it is located in birds’ skeletal systems [19]. The recommended ratio of calcium to phosphorus in the diet for bone health is 2:1 [20]. Dietary requirements for calcium and phosphorus in poultry diets depend on the type of poultry, age, and production phase. For example, the calcium requirements of pullets range from 0.92% to 2.25% and phosphorus around 0.40% to 0.45%, while broiler calcium requirements range from 0.75% to 0.95% and phosphorus from 0.30% to 0.45% on diet formulation [21]. Based on the analysis result, waste from chitin production still contains high levels of calcium and phosphorus. The waste from each stage has the potential to be a mineral supplement in animal feed.

**Table 2.** The amino acid component of chitosan processing waste.

No	Parameters	Unit	Neutral	Distillation	Demineralization	Deproteination
1	L-Alanine	mg/kg	1,118.67	237.425	<84.63	1,523.94
2	L-Arginine	mg/kg	<386.22	Not detected	Not detected	<386.22
3	L-Aspartic acid	mg/kg	794.995	406.1	<190.57	1,660.675
4	Glycine	mg/kg	1,115.145	384.485	164.965	1,735.785
5	L-Glutamic acid	mg/kg	1,738.405	507.055	<152.43	2,592.505
6	L-Histidine	mg/kg	<295.11	Not detected	Not detected	<295.11
7	L-Isoleucine	mg/kg	562.85	<170.99	Not detected	944.285
8	L-Leucine	mg/kg	483	228.79	Not detected	1,427.72
9	L-Lysine	mg/kg	550.35	Not detected	Not detected	808.415
10	L-Valine	mg/kg	816.435	200.81	<128.75	1,237.645
11	L-Phenylalanine	mg/kg	<476.07	<476.07	Not detected	959.83
12	L-Proline	mg/kg	671.985	231.105	<128.38	972.75
13	L-Serine	mg/kg	151.545	<149.74	Not detected	572.175
14	L-Threonine	mg/kg	192.55	<163.11	Not detected	183.705
15	L-Tyrosine	mg/kg	<608.01	Not detected	Not detected	737.835

### Amino acid results

The results of the analysis of the amino acid composition of waste from the chitin-making process can be seen in Table 2. Waste at each stage of chitin making and the neutral mixture of deproteinization and demineralization waste show different results. Of the 15 types of amino acids, there are 8 essential amino acids and seven essential amino acids. The eight essential amino acids include L-Arginine, L-histidine, L-Isoleucine, L-leucine, L-Lysine, L-Phenylalanine, L-Valine, and L-Threonine. The seven non-essential amino acids include L-Serine, L-Alanine, L-Glutamic acid, L-Proline, L-Tyrosine, Glycine, and L-Aspartic acid. The results of the analysis of 15 types of amino acids showed that complete amino acid content was found in deproteination process waste and neutral mixtures. Each solution has a different amino acid composition. Amino acids are one of the essential nutrients for preventing oxidative stress and the immune system and maintaining normal physiological, biochemical, and homeostatic mechanisms [22]. The highest amino acid content in deproteination and neutral mixture was L-Glutamic acid, namely 2,592.51 mg/kg and 1,738.41 mg/kg.

The amino acid L-Glutamic acid is a non-essential amino acid that is beneficial for health and productivity. L-Glutamine and L-Glutamic acid supplementation gave the best results (*p *< 0.05), increasing feed intake and body weight and reducing feed conversion rates in weaned pigs [23]. Glutamic acid supplementation can improve the performance and health of weaned pigs by increasing immune response, improving intestinal morphology, increasing digestive capacity, and increasing digestive tract microbial function [24]. The highest essential amino acid was found in the deproteination process, namely L-Leucine 1,427.72 mg/kg. The amino acid L-Leucine is an essential amino acid; therefore, livestock must get its supply from feed. L-Leucine plays an important role in the body’s metabolic system, cell function, enzyme activation, ATP formation, and insulin secretion from pancreatic islet cells [25]. Leucine plays an important role in fat metabolism and energy homeostasis. L-Leucine supplementation can help improve antioxidant status after heat stress in broiler chickens [26,27]. Chitosan production waste and neutral mixtures contain both essential and non-essential amino acids. This means that chitosan production waste could be used to make premixes.

## Conclusion

Each stage of the chitosan manufacturing process (demineralization, deproteination, and deacetylation) and the neutral mixture still contains bioactive such as crude protein, crude fat, carbohydrates, minerals (Ca, P, and K), as well as several types of essential and non-essential amino acids. These results indicate that chitosan processing waste has potential as a material for making premixes.

## List of abbreviations

BPS, Badan Pusat Statistik (Statistics Indonesia); CP: Crude Protein; CaCo_3_, calcium carbonate; HCl, hydrochloric acid; ICP-OES, inductively coupled plasma – optical emission spectrometry; NaOH, sodium hydroxide; PT: Perseroan Terbatas (Limited liability company); UPLC, ultra performance liquid chromatography.
